# *Staphylococcus aureus* phenol-soluble modulins have dispersal and anti-aggregation activity towards corynebacteria

**DOI:** 10.1128/jb.00183-25

**Published:** 2025-08-14

**Authors:** Joshua T. Huffines, Megan R. Kiedrowski

**Affiliations:** 1 Division of Pulmonary, Allergy and Critical Care, Department of Medicine, The University of Alabama at Birmingham 9968, Birmingham, Alabama, USA; University of Illinois Chicago, Chicago, Illinois, USA

**Keywords:** nasal colonization, aggregation, polymicrobial interactions, *Corynebacterium*, quorum sensing

## Abstract

*Staphylococcus aureus* is a common upper respiratory tract (URT) pathobiont with high carriage rates in the upper airway disease chronic rhinosinusitis (CRS). CRS is associated with increased prevalence and abundance of *S. aureus*, and *S. aureus-*secreted toxins are implicated in CRS pathogenesis. Conversely, in CRS disease, the abundance of non-pathogenic commensal species has been observed to decline, leading to microbial dysbiosis that contributes to persistent inflammation. Here, we investigated possible mechanisms *S. aureus* could employ to outcompete commensal organisms and contribute to establishing the *S. aureus-*dominant microbiome found in individuals with CRS. We performed a targeted screen to identify *S. aureus-*secreted factors that affect the growth and aggregation of a URT commensal bacterium, *Corynebacterium pseudodiphtheriticum*, which is negatively correlated with *S. aureus* in CRS. *S. aureus* cell-free conditioned media prevented *C. pseudodiphtheriticum* aggregation; however, anti-aggregation activity was significantly reduced in *S. aureus* mutants lacking a functional accessory gene regulator (agr) quorum-sensing system, phenol-soluble modulin (PSM) transporters, and the PSM toxin δ-toxin. Addition of purified recombinant δ-toxin peptide or a related PSM, PSMα3, inhibited *C. pseudodiphtheriticum* aggregation and induced dispersal of aggregates. Recombinant δ-toxin also reduced *C. pseudodiphtheriticum* adherence and aggregation on human nasal epithelial cells. PSMs are known to play a role in biofilm structure and remodeling in staphylococci, and here, we demonstrate that PSMs have activity against other bacteria. These results identify a novel mechanism by which *S. aureus* can disrupt the commensal lifestyle of microbes that inhabit the same upper respiratory niche via secreted PSM toxins.

**IMPORTANCE:**

Increased *Staphylococcus aureus* abundance and microbial dysbiosis are associated with the pathogenesis of chronic rhinosinusitis disease. Here, we show that *S. aureus* δ-toxin, a secreted phenol-soluble modulin (PSM) toxin, can inhibit the ability of commensal *Corynebacterium* species to aggregate, adhere to, and grow in association with human nasal epithelial cells. PSMs are known to play a key role in the *S. aureus* biofilm life cycle, regulating *S. aureus* biofilm structure and detachment; however, a role for these toxins in modifying biofilm and aggregate structures of other bacteria has not been previously demonstrated. These results suggest a potential mechanism for *S. aureus* to establish dominance in the upper respiratory tract microbiome in disease through direct antagonism of commensal microbes with PSM toxins.

## INTRODUCTION

The upper respiratory tract (URT) harbors numerous bacterial species that utilize a broad array of mechanisms to compete with one another for available resources (1, 2). Although many species can be identified in the URT, most occur at low abundance, and relatively few species account for most of the sequencing reads in URT microbiome analyses (3). Among the most abundant species are *Staphylococcus aureus* and members of the genus *Corynebacterium*. Multiple studies have found that these bacteria are negatively correlated with each other in the URT (3–6). This observation is particularly significant for chronic rhinosinusitis (CRS), a highly prevalent and costly disease of the URT (7–9). In CRS, overall URT microbial diversity is significantly reduced, and the resulting dominance established by pathogenic organisms is a significant contributor to disease pathogenesis (10–13). *Staphylococcus aureus* is significantly associated with CRS pathogenicity, with increased *S. aureus* carriage in CRS patients that correlates with disease severity and recurrence, toxin presence in tissue samples, and antibody specificity to well-known *S. aureus* virulence factors (14–17). Considering the association of *S. aureus* with URT disease and its negative correlation with *Corynebacterium* species, studies from two separate groups have explored potential mechanisms by which *Corynebacterium* may be able to inhibit *S. aureus* in the healthy airways (18, 19). Ramsey et al. (19) found that several species of *Corynebacterium* can limit *S. aureus* virulence by inhibiting staphylococcal quorum sensing via a similar mechanism of quorum-sensing interference that has been observed between competing strains of staphylococci, while Hardy et al. (18) discovered that some *Corynebacterium* strains can induce killing in *S. aureus* (19–21). Each of these mechanisms involves activation of the *S. aureus* accessory gene regulator (agr) system, the major quorum-sensing system in staphylococci that also regulates cell surface proteins and secreted virulence factor production (22).

The *S. aureus* agr system governs the switch between the staphylococcal biofilm lifestyle and acute virulence. Deactivation or inhibition of agr is associated with commensalism and reduced virulence, whereas agr activation promotes the production of secreted virulence factors and pathogenic behavior (23, 24). Agr activation occurs through sensing of the *S. aureus* auto-inducing peptide by its cognate membrane-localized histidine kinase, AgrC (22, 23). AgrC then phosphorylates the response regulator, AgrA, leading to transcription of the agr machinery, AgrABCD, and the primary downstream effector that governs toxin production, a regulatory RNA known as RNAIII (22, 23). Toxins regulated by the agr system have been thoroughly linked to *S. aureus* pathogenicity in CRS (9, 14, 17, 25–30). These include alpha hemolysin, leukocidins, proteases, and the phenol-soluble modulins (PSMs), short amphipathic peptides that have cytolytic properties as well as roles in *S. aureus* biofilm remodeling and dispersal due to their proposed surfactant-like properties that interact with plasma membranes. Several types of PSMs are encoded by *S. aureus*, including the α-PSMs in one operon, β-PSMs in another operon, and δ-toxin, which is encoded within RNAIII.

Considering the central role of agr in both CRS pathogenicity and polymicrobial interactions, a gap in knowledge exists regarding how *S. aureus* can overcome this inhibition to outcompete URT commensals and become dominant in the CRS microbiome in sinus disease. Most studies to date have examined the influence of other bacterial species on *S. aureus*, whereas if and how *S. aureus* has activity toward commensal competitors has not been well characterized (1). Previously, we examined interactions between *Corynebacterium* and *S. aureus* using air-liquid interface nasal epithelial cell co-culture to model URT colonization (31). Using a sequential model of infection, we observed that *S. aureus* was capable of colonizing human nasal epithelial cells (HNECs) despite the presence of *Corynebacterium pseudodiphtheriticum*. Furthermore, co-colonization with *S. aureus* led to an observable decrease in *C. pseudodiphtheriticum* aggregate formation on HNECs. This suggests to us that in CRS disease, where *S. aureus* is highly abundant in the inflamed URT, *S. aureus* may be capable of outcompeting *Corynebacterium* occupying the same niche.

Here, we identify a novel function for a CRS-associated *S. aureus*-secreted toxin that can directly alter *Corynebacterium* aggregation and adherence to human nasal epithelial cells. Utilizing a microscopy-based aggregation assay with a model commensal bacterium commonly found in the healthy URT, *Corynebacterium pseudodiphtheriticum*, we found that *S. aureus* secreted factors can inhibit *C. pseudodiphtheriticum* aggregation in a non-bactericidal manner. Next, utilizing the *S. aureus* Nebraska Transposon Mutant Library (32), we identified the secreted factor from *S. aureus* to be agr-regulated and dependent on the PSM transporter (PMT) complex. Further testing of *S. aureus* mutants lacking PSM genes, as well as addition of purified PSM peptides, confirmed the ability of PSMs to inhibit *C. pseudodiphtheriticum* aggregation and identified δ-toxin as the primary PSM responsible for this activity. Using an air-liquid interface nasal epithelial cell culture model to evaluate *C. pseudodiphtheriticum* colonization (31), we demonstrate the ability of exogenous δ-toxin to inhibit *C. pseudodiphtheriticum* adherence to the nasal epithelium. Taken together, this study characterizes a mechanism by which *S. aureus* can limit aggregation and induce dispersal of URT commensals with a CRS-associated toxin.

## MATERIALS AND METHODS

### Bacterial strains and culture

*Staphylococcus aureus* and *Corynebacterium* spp. strains used in this work are detailed in Table S1. Bacterial stocks were stored at −80°C in 20% glycerol. Bacteria from frozen stocks were streaked onto brain-heart infusion (BHI) agar plates with antibiotics if they contained selective markers. Overnight cultures were inoculated with a single colony from agar plates. BHI broth was used for *in vitro* culture of all strains. *Corynebacterium* spp. cultures were incubated at 30°C with shaking at 200 RPM, while *S. aureus* strains were incubated at 37°C with shaking for 24 hours. *Corynebacterium accolens* culture media were supplemented with 0.05% Tween 80 (Acros Organics) for overnight cultures. Kanamycin (50 µg/mL, ThermoFisher) was added to tdTomato-expressing *C. pseudodiphtheriticum* agar plates and cultures for the maintenance of the pJOE7706.1-tdTomato plasmid. Transposon insertion mutants from the Nebraska Transposon Mutant Library were grown with 50 µg/mL erythromycin (ThermoFisher) on BHI agar. Transposon mutant strains from the Network on Antimicrobial Resistance in *Staphylococcus aureus* and *Corynebacterium amycolatum* were obtained from BEI Resources, NIAID, NIH.

### Aggregation inhibition assay

*S. aureus* strains inoculated into 5 mL BHI broth were incubated for 24 hours at 37°C with shaking at 200 RPM. Cultures were centrifuged at 3,000 RCF for 5 minutes to pellet bacteria. Culture supernatant was filter sterilized using a 0.22 µm-pore syringe filter (Fisher Scientific) to obtain *S. aureus* cell-free conditioned medium (CFCM). *C. pseudodiphtheriticum* with pJOE7706.1-tdTomato plasmid was diluted into BHI broth to an *A*_600_ of 0.002 along with 0.2 mM isopropyl β-D-1-thiogalactopyranoside (IPTG; Sigma-Aldrich) to induce tdTomato expression and 100 µg/mL kanamycin. CFCM from *S. aureus* was diluted with BHI broth to 2× concentration of the desired volume/volume percentage. Similarly, for the addition of recombinant PSMs, antibiotics, or Tween 80, stock concentrations were diluted in BHI to 2× of the desired concentration. A volume of 50 µL of the cell-free conditioned medium was added to an untreated 96-well microtiter dish (Corning), followed by 50 µL of 2× *C*. *pseudodiphtheriticum*/IPTG mixture and kanamycin. The plate was incubated at 30°C without shaking for 20 hours. For other strains of *Corynebacterium*, the assay was performed without kanamycin or IPTG (to induce tdTomato expression from the pJOE7706.1-tdTomato plasmid), with 16 hours of growth in BHI broth. Imaging was performed using a Nikon Eclipse Ti2 widefield using a Plan Apo VC 20× Air N2 lens. Images were acquired from the center of each well (±300 µm) to limit variation of brightfield intensity due to edge effect. To obtain *C. pseudodiphtheriticum* colony-forming unit (CFU) counts, samples were mixed by vigorous pipetting and serially diluted in 0.1% Triton X-100 in PBS to help resolve aggregates, then plated to BHI agar to enumerate viable bacteria.

### Image analysis and quantification

Analysis of images was done using the Nikon NIS-Elements AR software package (version 5.42.02 Build 1801). Quantification of aggregate size was determined using the Object Counting tool in NIS-Elements. Minimum intensity projections of the z-stack were created, followed by auto-thresholding of the brightfield intensity using the Otsu Original method. Raw data from the object-counting tool were exported as a tab-delimited text file prior to analysis. Text files were imported into RStudio (version 2024.04.0 Build 735 “Chocolate Cosmos” Release). Area values below 1 were excluded to reduce exceedingly small values and allow for log2 transformation. The mean aggregate area for an individual well was calculated and averaged among wells for that group using base R.

### Live imaging microscopy

Time-lapse microscopy was performed under similar conditions as listed above, with the following modifications. Glass bottom Ibidi µ-Slide dishes were used for compatibility with the Nikon Perfect Focus System. Due to the increased surface area of the wells, 300 µL was the final volume as opposed to 100 µL. For dispersal with recombinant peptides, *C. pseudodiphtheriticum* was grown for 20 hours prior to the addition of synthesized peptide or phosphate-buffered saline, serving as the vehicle control. Incubation continued for 4 hours after the addition of the synthetic peptide.

### Cell-free conditioned medium treatments

Heat treatment, as well as proteinase-K incubation/inactivation, utilized thermocyclers for temperature consistency and replicability. BHI broth, serving as a treatment control and *S. aureus* cell-free conditioned medium (100%, vol/vol), was heated to 98°C for 50 minutes, followed by 10 minutes at 4°C. Protease treatment used 100 µg/mL proteinase-K (New England Biosciences) with a 50-minute incubation at 56°C, followed by a 10-minute heat-inactivation step at 98°C. For size fractionation, Amicon 3 kDa cutoff centrifugal filters (Millipore-Sigma) were used. Flow through was used directly in the assay, whereas the concentrate was diluted in fresh BHI to the original volume, followed by centrifugation at 13,000 RCF for the removal of precipitates.

### Human nasal epithelial cell co-culture assays

Cell culture experiments were conducted as described previously with slight modification (31). The RPMI 2650 human nasal epithelial cell line obtained from ATCC (ATCC CCL-30) was cultured in Eagle’s minimal essential medium (MEM; Corning) supplemented with L-glutamine (Gibco), 10% fetal bovine serum (Gibco), Pen-Strep (Gibco), and Plasmocin (Invivogen). Trypsin (Corning) was used to lift nasal cells prior to seeding in 6.5 mm transwell filters (CellTreat) pre-coated with Vitrogen plating medium as described previously (31). Transwell filters were incubated for 1 week prior to removing apical media for the transition to air-liquid interface. Nasal cells were then maintained at an air-liquid interface for 1 week prior to use in co-culture assays. Colonization with *C. pseudodiphtheriticum* was tested by inoculating the apical surface of ALI cultures with bacteria at an *A*_600_ of 0.1 in serum- and antibiotic-free MEM. For colony-forming unit assays, a 100 µL wash with MEM was used to remove non-adherent bacteria from the cell surface followed by 15 minutes of orbital shaking at 200 rpm with 100 µL of MEM containing 0.1% Triton X-100. Nasal cells were then scraped from the surface of the transwell filters and briefly vortexed to ensure dissociation of bacteria prior to serial dilution and viable colony-forming unit counts. For microscopy, transwell filters were fixed with 4% paraformaldehyde (Electron Microscopy Sciences) overnight at 4°C. Filters were then washed with Dulbecco’s phosphate-buffered saline (Sigma-Aldrich), followed by permeabilization in PBS with 0.1% Triton X-100 (Bio-Rad). DNA was stained with Hoechst 33342 (Invitrogen) prior to mounting with ProLong Gold (Invitrogen). Fluorescence microscopy was performed using a Plan Fluor 40× Oil DIC H N2 lens with Type B non-drying immersion oil (Cargille Laboratories) on a Nikon Eclipse Ti2 microscope as described above.

For assays evaluating *S. aureus* CFCM, 10% (vol/vol) CFCM was added concurrently with *C. pseudodiphtheriticum* inocula for adherence assays. Viable colony-forming units were collected and enumerated on BHI agar plates after 1 or 6 hours of co-culture with HNECs as indicated. For assays evaluating the effects of recombinant PSM peptides, *C. pseudodiphtheriticum* was inoculated with 5 µg/mL δ-toxin or vehicle control. Cytotoxicity was evaluated using the CytoTox 96 Non-Radioactive Cytotoxicity assay (Promega), which measures lactate dehydrogenase (LDH) release. Basolateral media were collected at the specified experimental endpoint and stored at −80°C. Values from a media-only control were subtracted from experimental values to remove background. A positive lysis control was collected through the addition of 10× lysis solution to the apical side of a vehicle-treated HNEC ALI culture for 30 minutes after the experimental endpoint. Experimental values were then normalized to the positive lysis measurement to yield the percentage of cytotoxicity.

### Recombinant peptide assays

Recombinant PSM⍺3 and δ-toxin (IBT Bioservices) were stored at −80°C. Prior to use in assays, the peptides were thawed in a 37°C water bath. Peptides were gently mixed and diluted into BHI broth for aggregation assays or MEM for HNEC co-culture assays at a 2× concentration. Control groups for these assays received an equal volume of vehicle, either MilliQ water for PSM⍺3 or phosphate-buffered saline for δ-toxin.

### Resazurin assay

Microtiter plates were set up similarly to the aggregation assay (see above) without IPTG or kanamycin added to the inoculum. Each test condition had a matching set of control wells without bacteria for normalization. Ten microliters of 0.22 µm-filtered 0.02% (wt/vol) resazurin salts (Thermo Scientific) was added to each well. The plate was incubated at 30°C in a Tecan Spark plate reader for 20 hours, with fluorescence readings taken every 30 minutes (excitation: 530 nm; emission: 590 nm). Values from control wells were subtracted from the experimental groups, resulting in the normalized values shown.

### Statistical analysis

GraphPad Prism (GraphPad Software; version 10.2.3 Build 347) was used for statistical analyses of colony-forming unit assays and aggregation assays. Normality of data was assessed with QQ-plots, probability of Gaussian distribution, and Shapiro-Wilk test. For one-way ANOVA tests, the Brown-Forsythe test was used to evaluate the homogeneity of variance, and the Shapiro-Wilk test was used to check the normality of residuals. Dunnett’s multiple comparisons test was used for *post hoc* analysis. For paired *t*-tests, the Shapiro-Wilk test was used to verify the normality of residuals. Error bars denote standard deviation. The lines shown on the graphs for individual groups represent the mean.

## RESULTS

### *Staphylococcus aureus* secreted factors inhibit *Corynebacterium* aggregation

**Fig 1:**
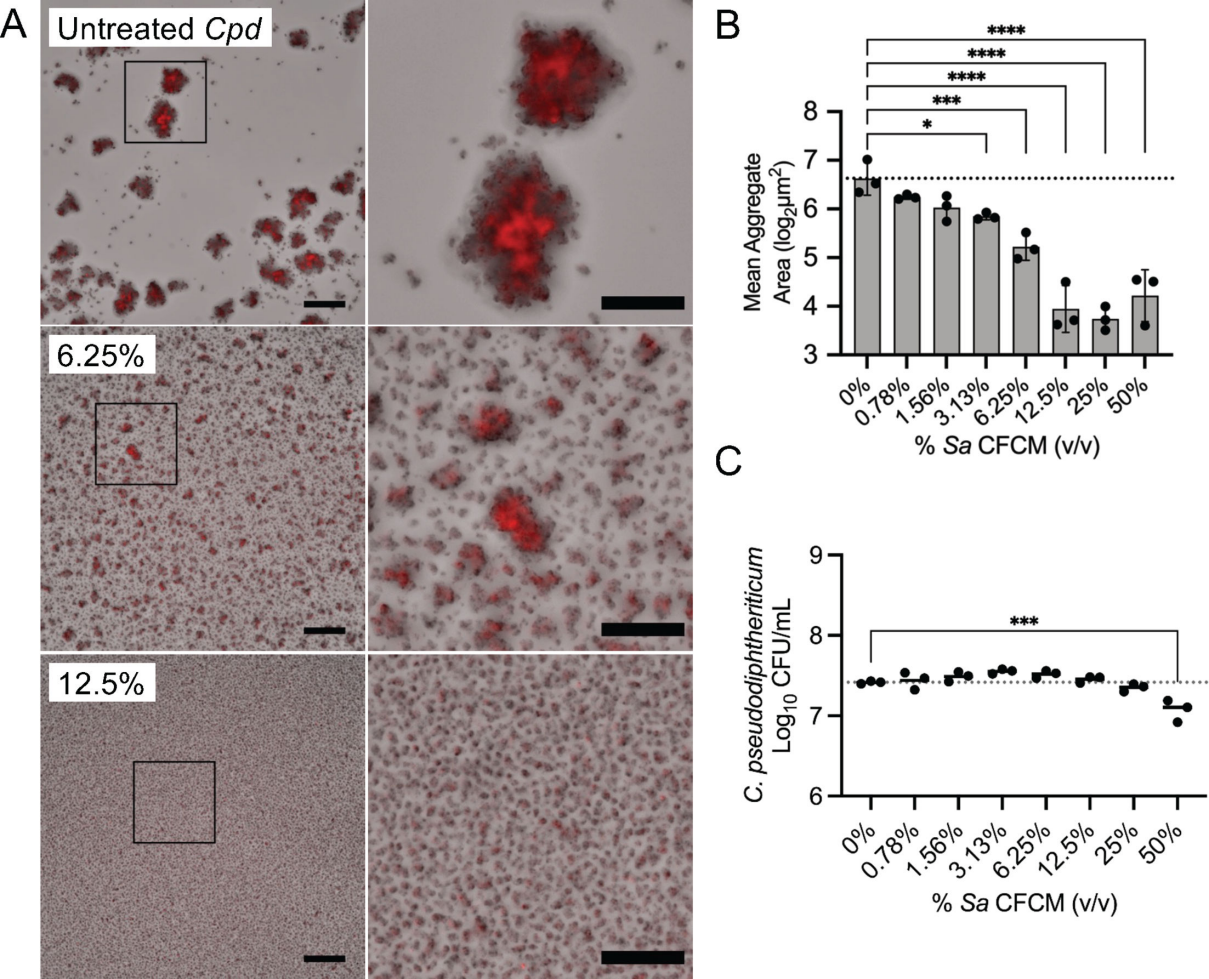
*Staphylococcus aureus* secretions inhibit *Corynebacterium* aggregate formation. (**A**) tdTomato *C. pseudodiphtheriticum* grown in brain-heart infusion broth for 20 hours with increasing concentrations of *S. aureus* CFCM prepared from *S. aureus* cultures grown for 24 hours at 37°C. Magnification (8×) of the inset is shown to the right. Images are representative of three biological replicates with three to six technical replicates each. Scale = 80 and 40 µm for 8× magnification. (**B**) Mean aggregation size for images in panel A. *n* = 3 biological replicates with three to six technical replicates each. (**C**) Colony-forming units of *C. pseudodiphtheriticum* aggregates disrupted with 0.1% Triton paired with A and B. *n* = 3 biological replicates with three technical replicates each. Statistics: one-way ANOVA with Dunnett’s multiple comparisons test. *P < 0.05, ***P < 0.001, and ****P < 0.0001.

We recently observed that *Corynebacterium* sinus isolates, including *C. pseudodiphtheriticum*, grew more slowly *in vitro* compared to *S. aureus* (31). Therefore, to test if *S. aureus* affects commensal bacterial growth or behavior independently of competition for nutrients in co-culture, we first examined the effects of *S. aureus*-secreted factors on *C. pseudodiphtheriticum* growth as a model URT commensal species using cell-free conditioned medium (*Sa* CFCM) prepared from stationary-phase *S. aureus* cultures. Using a tdTomato-expressing *C. pseudodiphtheriticum* strain (31), we observed that *C. pseudodiphtheriticum* forms large aggregates in liquid culture; however, there was a substantial decrease in the size of *C. pseudodiphtheriticum* aggregates when grown in the presence of *Sa* CFCM (Fig. 1A). We developed a high-throughput, quantifiable microscopy-based aggregation assay to further characterize this phenotype. Average *C. pseudodiphtheriticum* aggregate size was significantly reduced when grown in the presence of *Sa* CFCM, even at levels below 5% (vol/vol) (Fig. 1B). We enumerated *C. pseudodiphtheriticum* CFUs to determine if *Sa-*secreted factors in CFCM affected *C. pseudodiphtheriticum* viability. To help resolve *C. pseudodiphtheriticum* aggregates and accurately determine CFU, serial dilutions were performed in a mild detergent (0.1% Triton X-100) using a protocol previously established to enumerate bacteria in biofilms formed on airway epithelial cells (31, 33, 34) with vigorous pipetting. We observed that *Sa* CFCM did not significantly impact *C. pseudodiphtheriticum* growth, even when added at up to 25% (vol/vol) to *C. pseudodiphtheriticum* cultures (Fig. 1C). *C. pseudodiphtheriticum* colony-forming units were only observed to decrease from untreated control cultures in the presence of 50% (vol/vol) *Sa* CFCM, likely attributable to lack of nutrients. Using live imaging, we assessed whether *C. pseudodiphtheriticum* was actively growing as aggregates and found that aggregate area steadily increased at a linear rate over 20 hours (Fig. S1; Movie S1).

**Fig 2:**
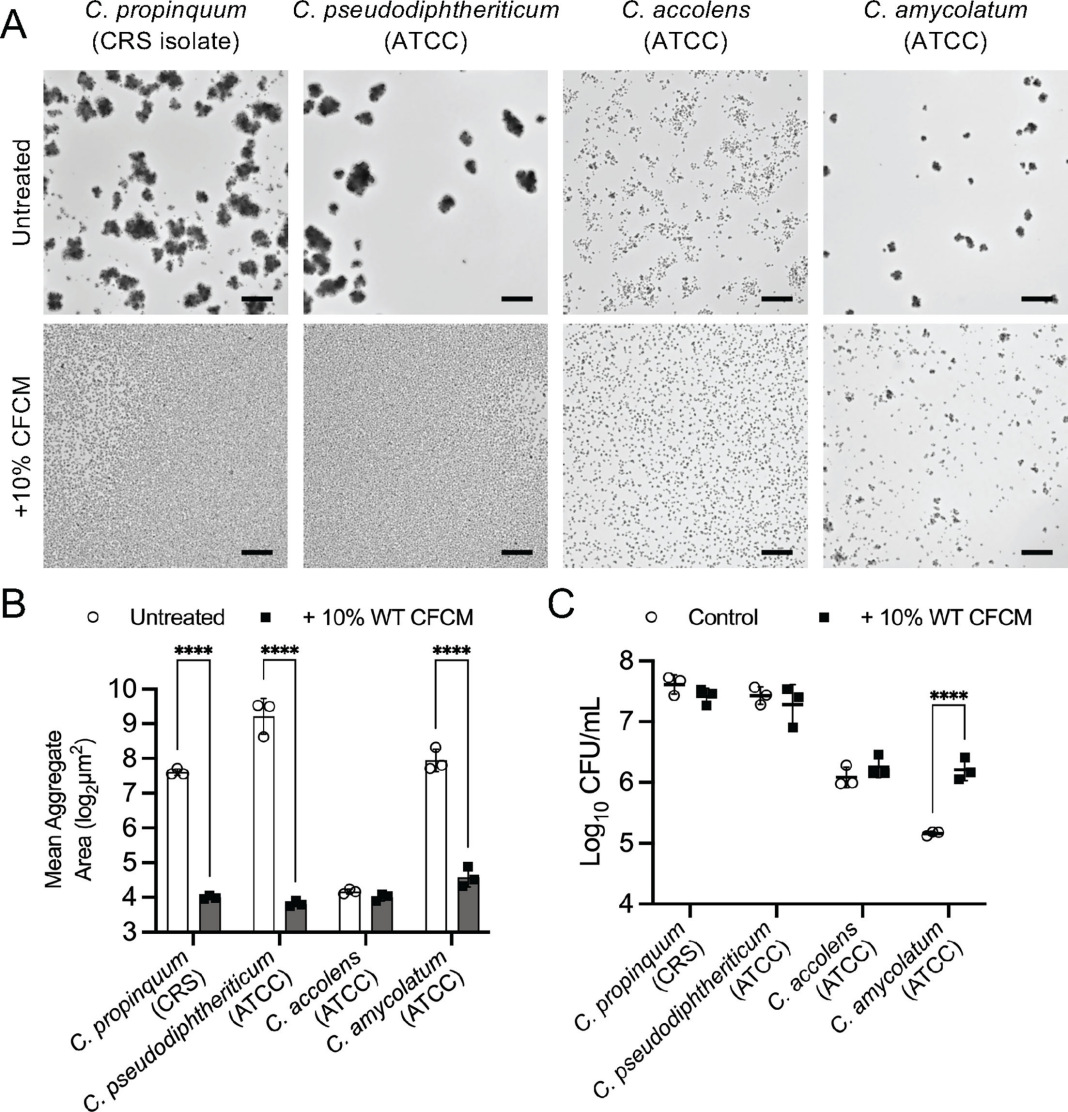
*Staphylococcus aureus* secretions inhibit *Corynebacterium* aggregation across species. (**A**) *Corynebacterium* species grown for 16 hours in BHI broth with 10% BHI or *S. aureus* CFCM prepared from *S. aureus* cultures grown for 24 hours at 37°C. Images are representative of three biological replicates with six technical replicates each. (**B**) Quantification of mean aggregate area from the images shown in panel A. Two-way ANOVA with Sidak’s multiple comparisons test. ****P < 0.0001. (**C**) Colony-forming units paired with A and B. *n* = 3 biological replicates with three technical replicates each. Statistics: one-way ANOVA with Dunnett’s multiple comparisons test. Two-way ANOVA with Sidak multiple comparisons test. ****P < 0.0001.

We next examined whether the ability of *Sa* CFCM to prevent *Corynebacterium* aggregation applied to other *Corynebacterium* species and strains (Fig. 2). Microscopy showed that a CRS clinical isolate of *Corynebacterium propinquum*, an ATCC strain of *Corynebacterium pseudodiphtheriticum*, and a skin isolate of *Corynebacterium amycolatum* all formed large aggregates when cultured in BHI broth, whereas a *Corynebacterium accolens* isolate formed aggregates with a substantially smaller area (Fig. 2A and B). The three strains that formed large aggregates had significantly lower average aggregate area when grown in the presence of 10% (vol/vol) *Sa* CFCM, similar to what we observed with our model CRS *C. pseudodiphtheriticum* strain (Fig. 2B). These strains also showed no defect in growth in the presence of *Sa* CFCM as measured by colony-forming unit counts (Fig. 2C). However, the average aggregate size of *C. accolens* was not significantly affected by *Sa* CFCM, likely attributable to the comparatively small aggregate size in the untreated group (Fig. 2A and B). *C. amycolatum*, which lacks an outer mycolic acid-rich mycomembrane found in most *Corynebacterium* species (35), was the only species found to have increased growth in the presence of *Sa* CFCM (Fig. 2C).

### Secreted proteins from *Staphylococcus aureus* inhibit *Corynebacterium pseudodiphtheriticum* aggregation

**Fig 3:**
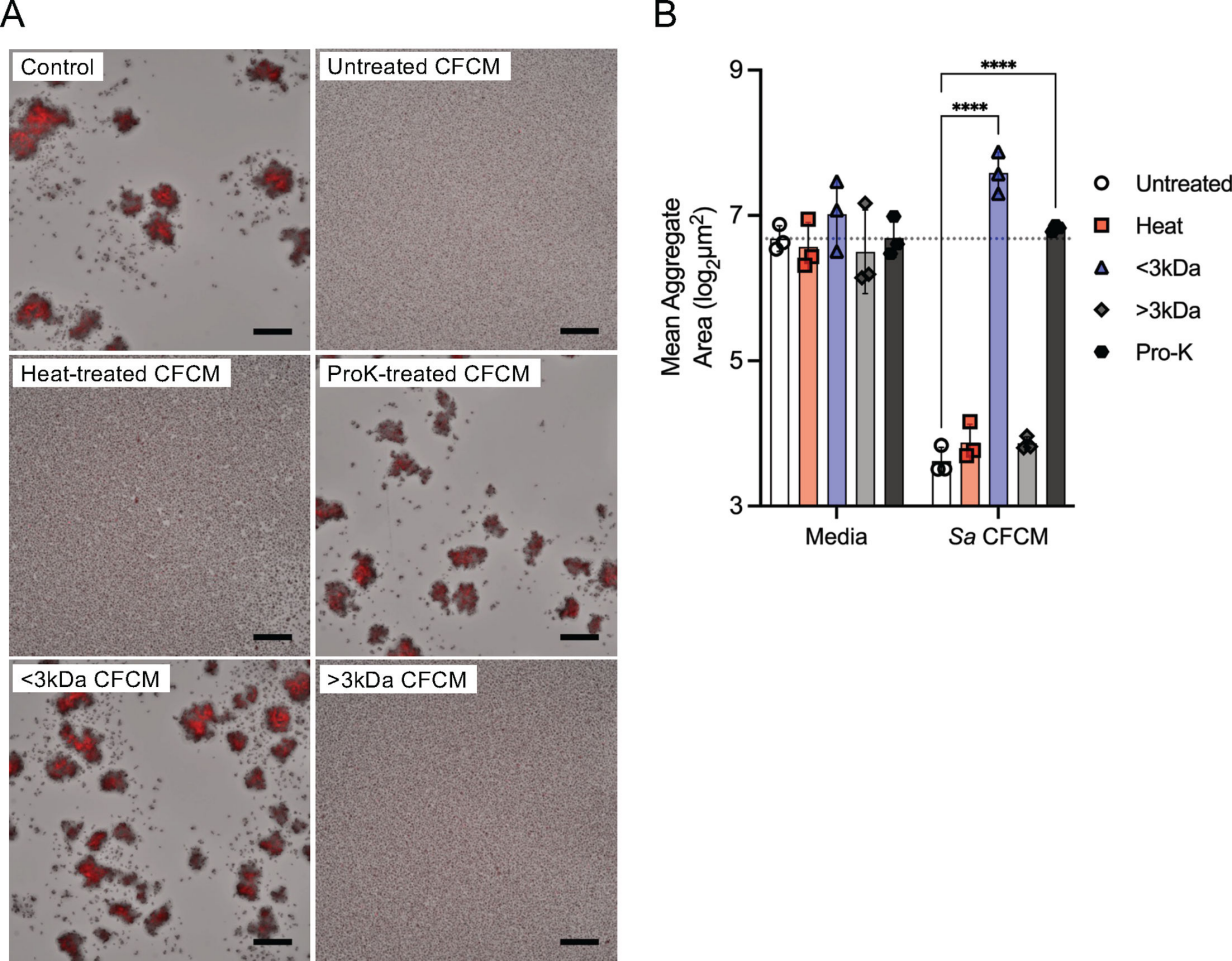
*Staphylococcus aureus* secreted proteins inhibit *Corynebacterium* aggregate formation. (**A**) tdTomato *C. pseudodiphtheriticum* grown for 20 hours in BHI broth with 25% BHI or *S. aureus* CFCM that was untreated, heated at 98°C for 1 hour, incubated with 100 µg/mL proteinase-K, filtered using a 3 kDa cut-off filter, or concentrated in the filter and diluted to the original concentration. *n* = 3 biological replicates with three technical replicates each. Scale bar = 80 µm. (**B**) Mean aggregation size for the images in panel A. *n* = 3 biological replicates with three technical replicates each. Two-way ANOVA with Dunnett’s multiple comparisons test (****P < 0.0001).

To characterize the nature of the *S. aureus-*secreted factor preventing *C. pseudodiphtheriticum* aggregation, we performed a series of treatments on *S. aureus* USA300 CFCM prior to using it in our aggregation assay at the highest percentage that did not significantly reduce *Corynebacterium* growth (Fig. 3). Heat-treated *Sa* CFCM retained the ability to inhibit *C. pseudodiphtheriticum* aggregation and reduce aggregate size. Hypothesizing that *S. aureus*-derived metabolites or small molecules could be influencing *C. pseudodiphtheriticum* aggregation, we tested *Sa* CFCM passed through an Amicon centrifugal filter with a 3 kDa cutoff and found that aggregate size was no longer affected, resembling untreated *C. pseudodiphtheriticum*. The addition of the *Sa* CFCM fraction retained in the centrifugal filter (diluted to the original volume to limit the effects of concentration) led to a significant reduction in aggregate size, as observed in the presence of untreated *Sa* CFCM. *Sa* CFCM treated with proteinase-K, followed by heat inactivation, lost the ability to inhibit *C. pseudodiphtheriticum* aggregation, resulting in aggregates similar in size to a media-only untreated control. Given this, we hypothesized that a secreted, heat-resistant *S. aureus* protein mediates aggregate inhibition.

### *Staphylococcus aureus* agr-regulated phenol-soluble modulins inhibit *C. pseudodiphtheriticum* aggregation

**Fig 4:**
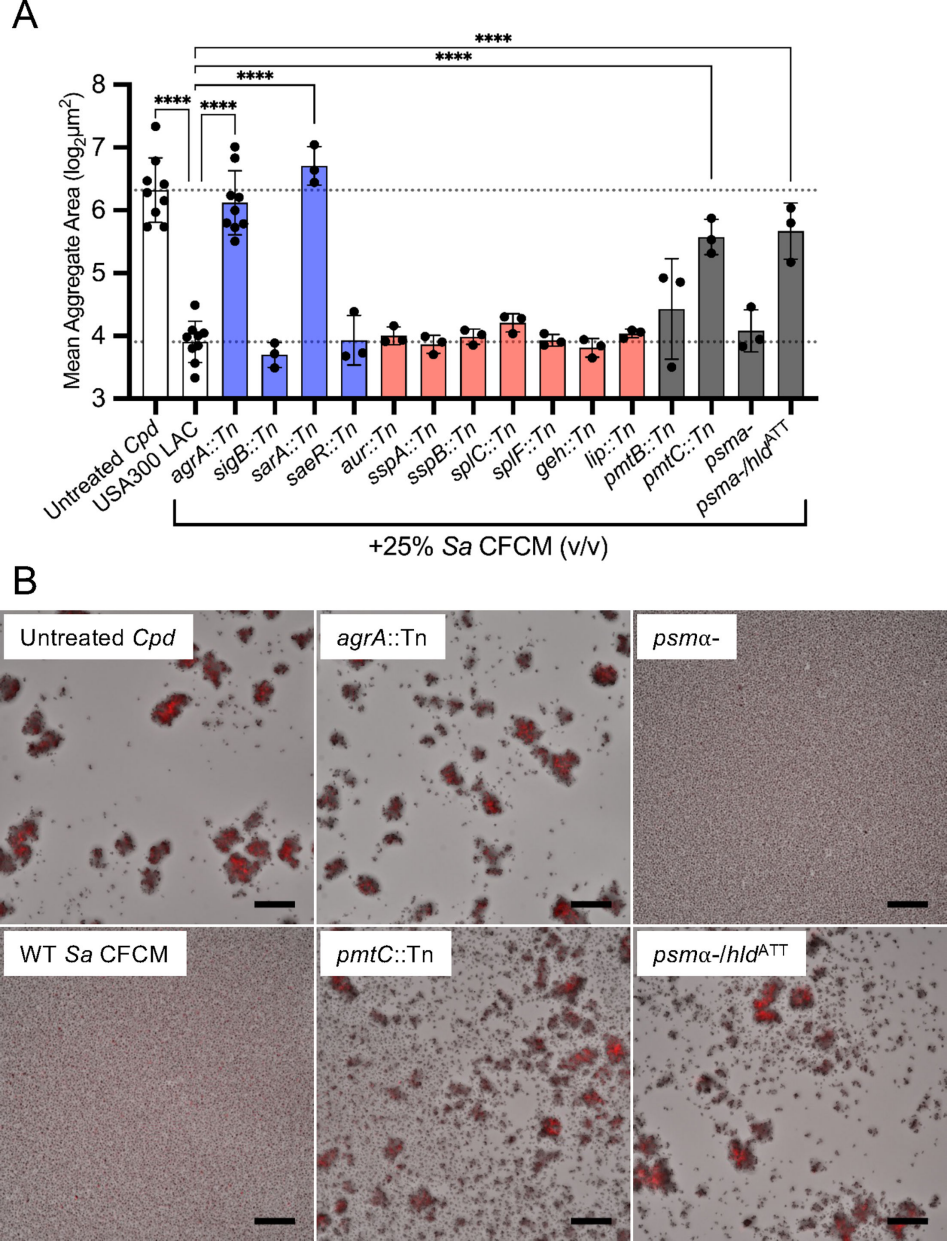
*S. aureus hld* is required to inhibit *Corynebacterium* aggregation. (**A**) Mean aggregate size quantification of tdTomato *C. pseudodiphtheriticum* exposed to 25% *S*. *aureus* CFCM from USA300 LAC 13c, transposon insertion mutants, or PSM-deficient strains for 24 hours. *n* = 3–9 biological replicates with three to six technical replicates each. One-way ANOVA with Dunnett’s multiple comparisons test. ****P < 0.0001. (**B**) Microscopy images from mutant screens as seen in panel A. Images are representative of three biological replicates with three to six technical replicates each. Scale = 80 µm.

The results of CFCM treatments suggested that the secreted *S. aureus* anti-aggregation factor capable of targeting other commensal species was a heat-stable protein larger than 3 kDa. We utilized the Nebraska Transposon Mutant Library (32) to conduct a targeted screen to identify genes involved in the regulation, production, and secretion of this factor (Fig. 4). We began by screening mutants in well-characterized regulatory genes and identified the expression of *agrA*, encoding the response regulator for the agr quorum-sensing system in *S. aureus*, and *sarA,* encoding a positive regulator for agr quorum-sensing (36), were necessary to inhibit *C. pseudodiphtheriticum* aggregation (Fig. 4; blue bars). Hypothesizing that an agr-regulated secreted enzyme was responsible, we next tested CFCM from several transposon mutants with disruptions in genes encoding agr-regulated proteases, which are known to play a role in biofilm dispersal in *S. aureus* (37), and lipases, which we believed may alter the corynemycolic acids of the *Corynebacterium* cell wall (Fig. 4A; red bars). However, these mutants all retained anti-aggregation activity similar to wild-type *Sa* CFCM. We next tested mutants with transposon insertions in genes encoding phenol-soluble modulin transporter proteins, *pmtB* and *pmtC*, as PSMs are known to be heat-resistant, agr-regulated proteins (22, 38–40). The *pmtB* transposon insertion partially restored *C. pseudodiphtheriticum* aggregation, and CFCM from the *pmtC* transposon insertion significantly abrogated the inhibitory effects of *S. aureus* CFCM (Fig. 4A; gray bars). To further narrow down which PSMs were responsible, CFCM from an *S. aureus psm*α1-4 deletion strain, lacking α-type PSMs 1 through 4, and a *psm*α1-4 deletion strain encoding an additional start codon mutation (ATG > ATT) in the *hld* gene, which encodes the 5th α-type PSM, δ-hemolysin, was tested (41). Only the *psm*α1-4 *hld*ATT double mutant lost the ability to inhibit *C. pseudodiphtheriticum* aggregation (Fig. 4; gray bars).

**Fig 5:**
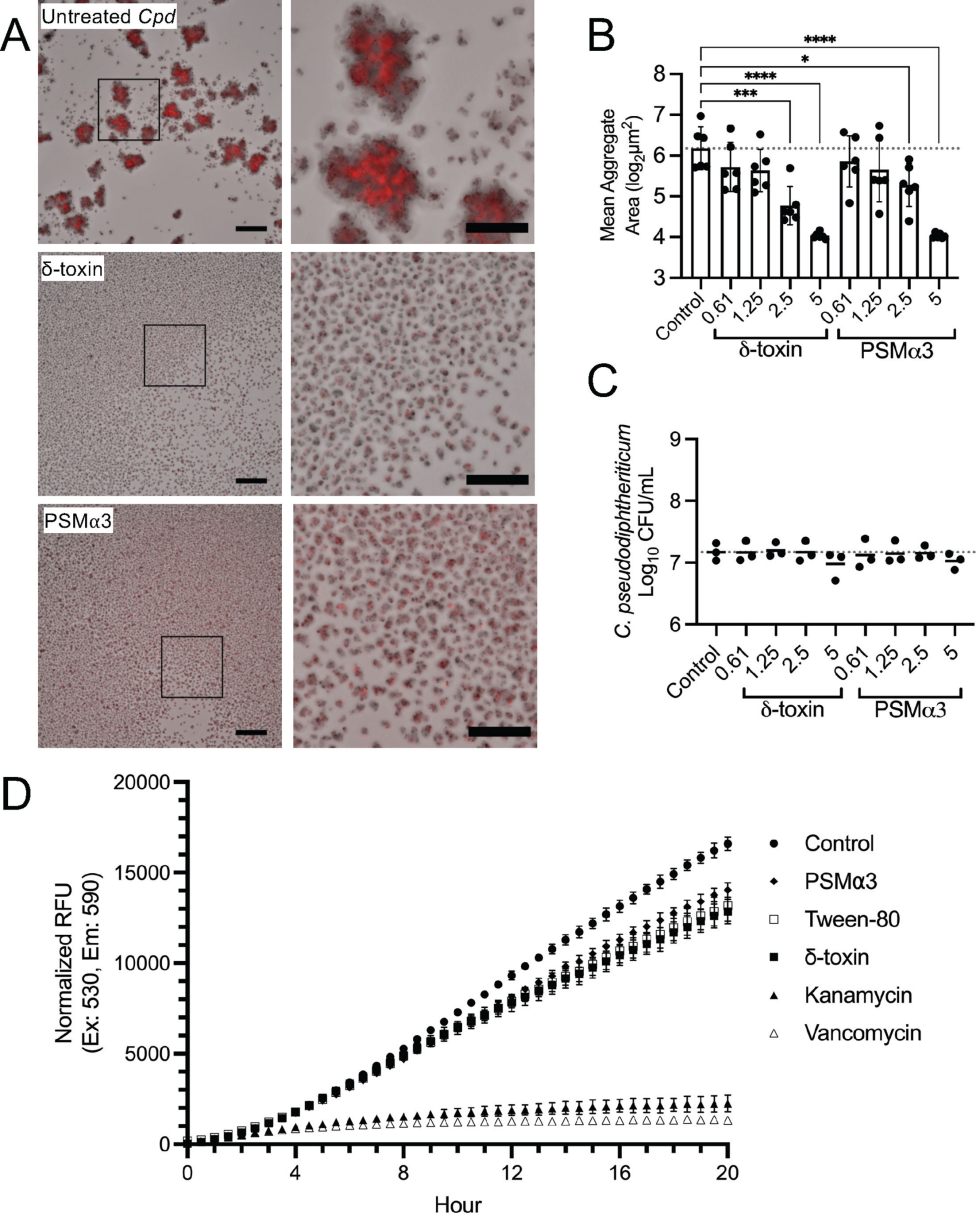
*S. aureus* phenol-soluble modulins inhibit *Corynebacterium* aggregation without killing. (**A**) tdTomato *C. pseudodiphtheriticum* grown for 20 hours with 5 µg/mL synthesized δ-toxin or PSMα3. Inset is shown on the right. Images are representative of three biological replicates with three technical replicates each. (**B**) Mean aggregate area quantification of microscopy images as shown in panel A. *n* = 6 biological replicates with three to six technical replicates each. One-way ANOVA with Dunnett’s multiple comparisons test. *P < 0.05, ***P < 0.001, and ****P < 0.0001. (**C**) Colony-forming units of *C. pseudodiphtheriticum* for A and B. *n* = 3 biological replicates with three technical replicates each. (**D**) *C. pseudodiphtheriticum* metabolic activity measured via resazurin reduction grown in the presence of 5 µg/mL δ-toxin, 5 µg/mL PSM⍺3, 0.01% Tween 80, 50 µg/mL kanamycin, and 50 µg/mL vancomycin. Data shown indicate relative fluorescence units (RFUs) with the values from matching media-only controls subtracted. *n* = 3 biological replicates with three technical replicates each.

To confirm the results observed with CFCM from *S. aureus* mutants, we next examined whether the addition of recombinant PSM peptides could inhibit *C. pseudodiphtheriticum* aggregation (Fig. 5). Production of the different types of PSMs encoded by *S. aureus* has been shown to vary substantially in culture (42); therefore, we tested the addition of δ-toxin and PSMα3 peptides individually. Addition of purified, recombinant PSM peptides ranging from 0.6 to 5 µg/mL significantly inhibited *C. pseudodiphtheriticum* aggregate formation for both recombinant δ-toxin and PSMα3 in a dose-dependent manner (Fig. 5A and B). Peptide concentrations evaluated were calculated to be equivalent to concentrations that would be present in up to 10% (vol/vol) *Sa* CFCM, based on previous measurements of PSMs generated in overnight stationary-phase *S. aureus* cultures (42). Neither recombinant δ-toxin nor PSMα3 affected *C. pseudodiphtheriticum* viability, determined by plating viable CFU after exposure to recombinant PSMs (Fig. 5C). Since the use of recombinant peptide avoids confounding nutrients in *Sa* CFCM, we also used a resazurin assay to measure the generation of bacterial NADH/NADPH through the reduction of resazurin to resorufin (43) to examine if the metabolism of *C. pseudodiphtheriticum* was affected in the presence of PSMs (Fig. 5D). The reduction of resazurin with the addition of PSMs resembled *C. pseudodiphtheriticum* grown with 0.05% Tween 80, a common addition to culture media to support the growth of actinobacteria, and which we observed can also inhibit aggregate formation (Fig. S2). We then tested whether δ-toxin is capable of dispersing pre-formed *C. pseudodiphtheriticum* aggregates using time-lapse microscopy (Fig. S3; Movie S2). Compared to the untreated control, *Corynebacterium* aggregates were disassembled by the addition of recombinant δ-toxin, with substantial disruption occurring within 2 hours. While significant loss of tdTomato fluorescence was observed, because we did not find reduced *C. pseudodiphtheriticum* viability with δ-toxin exposure (Fig. 5C), we predict that this can be attributed to reduced fluorescent signal in smaller aggregates and individual bacteria as a result of dispersal. This is likely compounded by photobleaching due to the increased frequency of imaging at 15-minute intervals.

A previous study by Su et al. (44) quantified δ-toxin production in several *S. aureus* strains, including well-known laboratory strains. To determine whether the production of δ-toxin correlates with the inhibition of *C. pseudodiphtheriticum* aggregation, we screened *S. aureus* strains from our collection that were included in that study (Fig. S4). We used *S. aureus* strains Mu50, N315, and 502A, representing *S. aureus* strains that are incapable of producing δ-toxin, produce lower levels of δ-toxin, and produce higher levels of δ-toxin, respectively. Notably, there was no significant change in *C. pseudodiphtheriticum* mean aggregate area in the presence of CFCM from Mu50 and N315 compared to the untreated control, whereas CFCM from *S. aureus* strain 502A, known to produce high levels of δ-toxin, significantly reduced aggregation (Fig. S4B). Viability of *C. pseudodiphtheriticum* was largely unaffected, except for a modest decrease with N315 CFCM (Fig. S4C).

### *Staphylococcus aureus* phenol-soluble modulins alter *Corynebacterium pseudodiphtheriticum* adherence to nasal epithelial cells

**Fig 6:**
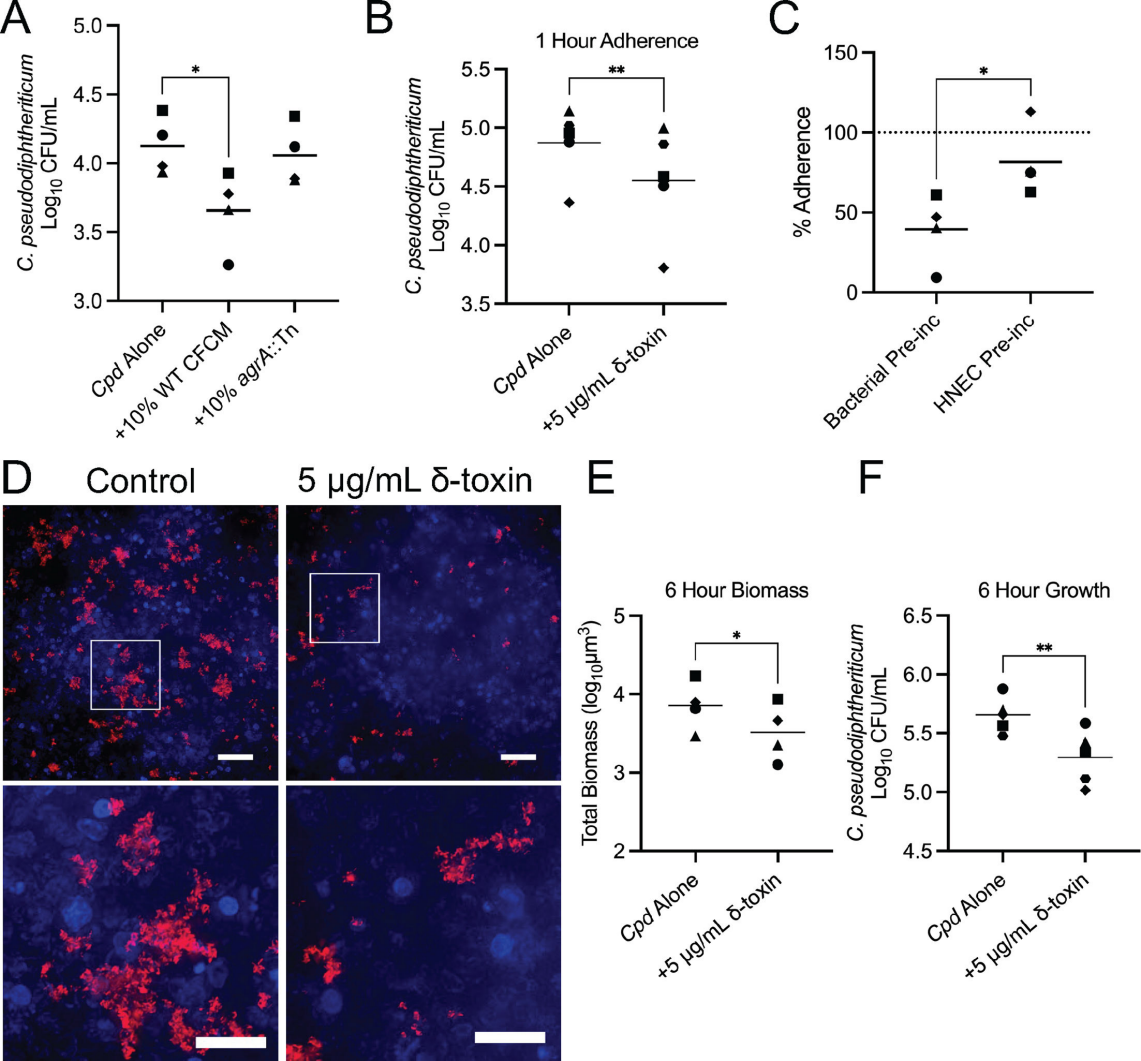
δ-toxin reduces *Corynebacterium* adherence to nasal epithelial cells. (**A**) Colony-forming units of *C. pseudodiphtheriticum* attached to human nasal epithelial cells after a 1-hour co-incubation with 10% *S*. *aureus* CFCM. *n* = 4 matched biological replicates. One-way ANOVA with Dunnett’s multiple comparisons test. *P < 0.05. (**B**) Colony-forming units of *C. pseudodiphtheriticum* co-incubated with δ-toxin on HNECs for 1 hour. *n* = 5 matched biological replicates. One-tailed paired *t*-test. **P < 0.01. (**C**) Adherence of *C. pseudodiphtheriticum* to HNECs following a 1-hour pre-incubation of 5 µg/mL δ-toxin with the bacteria or HNECs. Data are normalized to the untreated control denoted by dashed line. One-tailed *t*-test, *P < 0.05. *n* = 4 matched biological replicates. (**D**) Microscopy images (40× magnification; highlighted region shown below) of tdTomato-labeled *C. pseudodiphtheriticum* adherent to HNECs after a 6-hour co-incubation with 5 µg/mL δ-toxin. Scale bar = 40 µm; 20 µm for 8× magnified region. (**E**) Biomass volume quantification of images from panel D. One-tailed paired *t*-test, *P < 0.05. (E) Colony-forming units of *C. pseudodiphtheriticum* attached to HNECs after 1 hour with a 1-hour pretreatment of the bacteria or HNECs with 5 µg/mL δ-toxin. Two-tailed *t*-test, *P < 0.05. (**F**) Colony-forming units of *C. pseudodiphtheriticum* to HNECs after 6 hours of growth with or without 5 µg/mL δ-toxin. *n* = 5 matched biological replicates. One-tailed *t*-test, **P < 0.01.

We previously observed that *Corynebacterium* CRS clinical isolates could colonize and form large aggregates in a co-culture model using polarized, air-liquid interface HNEC cultures (31). Given our observations of the effects of *Sa* secreted factors on *Corynebacterium* aggregation *in vitro*, we next asked if *Sa* CFCM would be sufficient to limit *C. pseudodiphtheriticum* growth in association with HNECs. We observed that the addition of CFCM from WT *S. aureus* strains resulted in a significant reduction in *C. pseudodiphtheriticum* adherence to HNECs at 1 hour with WT CFCM, but not CFCM from the *agrA::Tn* strain (Fig. 6A), supporting our earlier observations in the *in vitro* aggregation assay (Fig. 4). We previously observed that *C. pseudodiphtheriticum* does not have significant cytotoxic effects on HNEC ALI cultures (31). To test if HNEC cytotoxicity due to *S. aureus* CFCM could be a potential factor affecting *C. pseudodiphtheriticum* adherence, we performed LDH release assays using basolateral medium from HNEC cultures treated with CFCM and observed no significant differences compared to controls (Fig. S5A). To examine whether *S. aureus* PSMs could interfere with *C. pseudodiphtheriticum* adherence and aggregate formation on HNECs, we inoculated *C. pseudodiphtheriticum* in the presence or absence of recombinant δ-toxin. Co-incubation of *C. pseudodiphtheriticum* with recombinant δ-toxin was sufficient to significantly decrease adherence at 1 hour (Fig. 6B). To confirm whether this effect from recombinant δ-toxin was targeting the *Corynebacterium* more so than the HNECs, 1-hour adherence to HNECs was examined after a pre-incubation of either the *Corynebacterium* or the HNECs with 5 µg/mL δ-toxin for 1 hour (Fig. 6C). Comparing these groups showed a significant decrease in adherence with the pre-incubated *Corynebacterium* over the pre-incubated HNECs. Extending the co-incubation with δ-toxin to 6 hours resulted in a significant decrease in *C. pseudodiphtheriticum* colonization of HNECs (Fig. 6D through F). Fluorescence microscopy of tdTomato-labeled *C. pseudodiphtheriticum* colonizing HNECs in the same conditions showed marked reduction in the formation of *C. pseudodiphtheriticum* aggregates in the presence of δ-toxin with lower total biomass as quantified through NIS-elements (Fig. 6D and E). Quantifying colony-forming units for *C. pseudodiphtheriticum* at the 6-hour time point demonstrated a significant decrease in *Corynebacterium* colonizing the HNECs when grown in the presence of δ-toxin (Fig. 6F). As with *S. aureus* CFCM, we did not observe that the addition of 5 µg/mL δ-toxin resulted in significant HNEC cytotoxicity following 1- and 6-hour incubations (Fig. S5B).

## DISCUSSION

While the detrimental role of *S. aureus* in URT disease is well established, and previous work has identified potential polymicrobial interactions that may occur between *S. aureus* and other URT colonizers (1, 2), few studies to date have assessed how *S. aureus* might mediate a transition from the healthy, microbially diverse URT to a diseased, pathogen-dominated URT via direct competition with commensal species that occupy the same niche. Here, we demonstrate a novel role for *S. aureus* δ-toxin, a CRS-associated virulence factor (14), in altering the commensal lifestyle and commensal-host interactions for a competing URT commensal colonizer, *Corynebacterium pseudodiphtheriticum*.

We demonstrated that the agr-regulated *S. aureus* toxins, δ-toxin and PSMα3, can prevent *Corynebacterium* aggregation, disperse pre-formed aggregates, and limit colonization of host nasal cells. Previous studies of *Corynebacterium* and *S. aureus* interactions showed that *Corynebacterium* can alter *S. aureus* behavior through inhibition of agr quorum sensing or by inducing killing in *S. aureus* strains with an active agr system (18, 19). This partially explains the negative correlation observed between *Corynebacterium* and *S. aureus* in the URT (3, 5, 6). However, when *S. aureus* does not have a functional agr system, its resulting gene expression is commensal-like and does not diminish its ability to colonize the upper respiratory tract, as numerous studies have demonstrated (19, 22, 23). Furthermore, *S. aureus* populations in the URT can be heterogeneous, with a portion being incapable of agr activation and therefore immune to *Corynebacterium*-induced killing (45). The ability of *Corynebacterium* species to inhibit or exploit the *S. aureus* agr system suggests that agr activation likely exerts a selective pressure on *Corynebacterium* co-existing with *S. aureus* in the URT. Biofilms and biofilm-like lifestyles, such as aggregation exhibited by *Corynebacterium*, are vital for some colonizing species (46). We speculate that the ability of *S. aureus* to disrupt this aggregation lifestyle in *Corynebacterium* (Fig. 1) could be the evolutionary pressure needed to conserve a method to inhibit *S. aureus*, specifically through the agr system that governs the production of PSM toxins. Further supporting this, we found that a functional agr system is necessary for *S. aureus* antagonism of *Corynebacterium* aggregation (Fig. 4). We saw that aggregation of three separate species of *Corynebacterium* (Fig. 2) was strongly inhibited by *S. aureus-*secreted factors, suggesting a broadly antagonistic relationship between commensal *Corynebacterium* species and *S. aureus*. In fact, a recent study showed that a secreted protease from a *C. pseudodiphtheriticum* strain can inhibit ⍺-hemolysin-independent hemolysis by *S. aureus* CFCM (47), which insinuates *Corynebacterium*-mediated degradation of PSMs that does not require the inhibition of agr quorum sensing as shown previously (19).

The significant association between *S. aureus* agr-regulated toxins and CRS pathogenesis is strong evidence that agr activation occurs in the URT in inflammatory disease and results in the production of δ-toxin (12, 17, 27, 48). Indeed, proteomics analysis of mucosal tissue samples from CRS patients found δ-toxin and multiple agr-related proteins (14). Our observation that agr-regulated δ-toxin can significantly disrupt *Corynebacterium* aggregation (Fig. 4 and 5) and its ability to colonize nasal epithelial cells (Fig. 6) offers a possible mechanism by which *S. aureus* can create not only an inflammatory environment but also an environment that is inhospitable for *Corynebacterium* and other commensal microbes reliant on aggregation or biofilm formation. As dysbiosis is a major component of CRS disease (10), and multiple URT commensals have mechanisms for antagonizing *S. aureus* (2, 49), it is reasonable that *S. aureus* is actively contributing to this process to outcompete other microorganisms. Supporting this, a recent study examined gene presence for agr-regulated toxins in *S. aureus* and found that while the presence of most genes ranged widely among CRS isolates, *hld* was conserved in 100% of isolates evaluated (50). Additionally, hypoxic conditions can increase agr activation and PSM production (51, 52), and the occluded sinuses in CRS are thought to be a microoxic environment (13, 53, 54). Interestingly, many *S. aureus* isolates from CRS have attenuation of the agr system resulting from adaptation in the chronically diseased airway environment. This has been shown to be mediated through the alternative sigma factor B (50, 55). Additionally, the SrrAB two-component system has been shown to inhibit agr activation in oxygen-depleted environments, which could lead to further reduction of agr activation in the occluded sinuses in CRS (56). A prior study observed variable levels of *S. aureus* agr activation in the presence of representative sinus microbial communities cultured anaerobically, with cell-free supernatants from some communities leading to heightened agr activation under anaerobic conditions (53). Collectively, these studies portray a complex relationship between the URT environment and agr activation. While *hld* is widely conserved, the production of δ-toxin can vary greatly between strains (44). Our own data support this, as multiple *S. aureus* strains were not capable of preventing *Corynebacterium* aggregation, specifically Mu50 and N315 (Fig. S4). This may be explained by the lack of δ-toxin production by Mu50 and low production by N315 in a study by Su and colleagues (44). In that study, the production of δ-toxin by *S. aureus* strain 502A was well above the average, consistent with our finding that 502A CFCM prevents *Corynebacterium* aggregation (Fig. S4). Although direct quantification of PSMs in the URT has not been demonstrated, a recent study measured PSM production in a murine air pouch infection model (42). A 1 mL lavage of the air pouch after 48 hours of infection contained, on average, >1 µg/mL δ-toxin, which supports that *S. aureus* is capable of producing high concentrations of δ-toxin during infection, consistent with the significantly higher quantities of δ-toxin and low levels of other α-type PSMs in *S. aureus* CFCM (42).

The complex role of δ-toxin in *S. aureus* biology limits the conclusions that can be drawn from CFCM studies. PSMs are known to regulate *S. aureus* biofilm structure, mediating detachment and microchannel formation, and PSM production can vary depending on the biofilm microenvironment (57). The dispersal ability of PSMs on *S. aureus* biofilms is believed to be mediated by their proposed activity as surfactants that can mediate spreading on wet surfaces (58). δ-toxin is believed to play a key role in the formation of *S. aureus* extracellular vesicles, which can contain numerous *S. aureus* products, as well as in amyloid fiber formation (42, 59). However, the other ⍺-type PSMs have been implicated in vesicle formation (60). Additionally, amyloid fibers have been found to be resistant to protease treatment (42), and the tertiary structure of some PSM amyloid fibers is sensitive to heat treatment (59). Our results implicate sensitivity to protease treatment and resistance to heat (Fig. 3), suggesting that amyloid fibers are not required for aggregation inhibition of *C. pseudodiphtheriticum*. While our findings show *hld* is necessary for aggregation inhibition (Fig. 4), the addition of recombinant PSMα3 was sufficient to inhibit aggregate formation in *Corynebacterium* (Fig. 5). We predict that the PSMα knockout strain (Fig. 4) retained the ability to inhibit *C. pseudodiphtheriticum* aggregation due to the significantly lower production of PSMα3 compared to δ-toxin that has been previously reported (42). Our data suggest a conserved ability of ⍺-type PSMs to inhibit *Corynebacterium* aggregation, which may stem from its previously reported surfactant-like activity (58). Although a previous study found bactericidal effects for proteolytic derivatives of PSMs (61), we did not observe significant growth limitation by *S. aureus* secretions (Fig. 1) and no such effect for either full-length PSM peptide at the highest levels tested (Fig. 5). While metabolism was mildly reduced in the presence of δ-toxin and PSM⍺3, addition of Tween 80 detergent had the same effect (Fig. 5), suggesting the aggregation inhibition by PSMs is from its surfactant-like properties, shown previously (58). Supporting this, we observed that Tween 80 also prevents *Corynebacterium* aggregation at levels as low as 0.01% (Fig. S2). Of note, we predict that the increased colony-forming units we measured in the presence of Tween 80 are likely a result of the ability of *Corynebacterium* species to metabolize Tween using lipases (47, 62). Bactericidal effects from PSM production may be limited to certain species, and it is possible that outer membranes in gram-negative bacteria and additional unique cell wall features of some gram-positive bacteria offer protection against PSM derivatives. Future studies will examine the mechanisms behind δ-toxin inhibition of *Corynebacterium* aggregation and ask if this anti-aggregation effect is specific for competition against *Corynebacterium* species or broadly effective against other URT-colonizing bacteria. Additionally, while we show that reduced adherence of *Corynebacterium* to epithelial cells likely stems from direct interactions between δ-toxin and *Corynebacterium* (Fig. 6C) and not an increase in epithelial cytotoxicity resulting from incubation with δ-toxin (Fig. S5), the ability of PSMs to modulate host airway epithelial physiology is not well characterized. PSMs have been shown to induce an immune response in skin epithelial cells at low concentrations mediated through the formyl-peptide receptor 2 (FPR2) (63). Airway epithelial cells are known to express FPR2 (64), yet no studies have examined whether recognition of *S. aureus-*derived PSMs occurs in the nasal epithelium and what the downstream ramifications would be on immune activation.

Collectively, these experiments lead us to hypothesize that *S. aureus* is capable of directly interfering with the *Corynebacterium* commensal lifestyle in the URT through the production of δ-toxin and alpha PSMs, contributing to the reduction in *Corynebacterium* abundance often observed in the diseased CRS microbiome. Several observations made by other groups support this hypothesis. *S. aureus* commensalism is linked to the inactivation of the agr system, either through genetic mutations, inhibition by *S. aureus* gene regulators, or external inhibition from competing bacteria (1, 2, 18, 19). During or prior to *S. aureus*-associated CRS pathogenesis, agr is activated, leading to the secretion of several virulence factors, including δ-toxin, which have been found in tissue samples obtained from subjects with CRS (14, 17, 27–30). We speculate that inhibition of *Corynebacterium* aggregation by δ-toxin provided the evolutionary pressure for *Corynebacterium* species to develop a yet unidentified method of inhibiting *S. aureus* agr activation. The ability of δ-toxin to reduce adherence of *C. pseudodiphtheriticum* to nasal epithelial cells and disperse pre-formed aggregates may promote immune clearance of *Corynebacterium* species and other commensal colonizers, as well as increase susceptibility to antibiotics, which will be investigated in future studies.

## SUPPLEMENTAL MATERIAL

Figure S1 jb.00183-25-s0001.tiff *Corynebacterium pseudodiphtheriticum* aggregate sizes increases over time.

Figure S2 jb.00183-25-s0002.tiff Tween 80 inhibits *Corynebacterium pseudodiphtheriticum* aggregation.

Figure S3 jb.00183-25-s0003.tiff *S. aureus* phenol-soluble modulins induce *Corynebacterium* dispersal.

Figure S4 jb.00183-25-s0004.tiff Inhibition of *Corynebacterium* aggregation is varied amongst *Staphylococcus aureus* strains.

Figure S5 jb.00183-25-s0005.tiff Toxicity of *Staphylococcus aureus* CFCM and recombinant δ-toxin towards air-liquid interface (ALI) human nasal epithelial cell (HNEC) cultures.

Supplemental legends and Table S1 jb.00183-25-s0006.pdf Supplemental legends for Fig. S1 to S5, and Movies S1 and S2. Table S1: Bacterial strains.

Movie S1 jb.00183-25-s0007.mp4

Movie S2 jb.00183-25-s0008.mp4 Timelapse of *C. pseudodiphtheriticum* culture exposed to δ-toxin after 20 hours of growth.

ASM does not own the copyrights to Supplemental Material that may be linked to, or accessed through, an article. The authors have granted ASM a non-exclusive, world-wide license to publish the Supplemental Material files. Please contact the corresponding author directly for reuse.
