# Supplementary figures and images for: Staphylococcus aureus phenol-soluble modulins have dispersal and anti-aggregation activity towards corynebacteria
Source: J Bacteriol. 2025 Aug 14;207(9):e00183-25. doi: 10.1128/jb.00183-25 (PMC12445096; doi:10.1128/jb.00183-25)

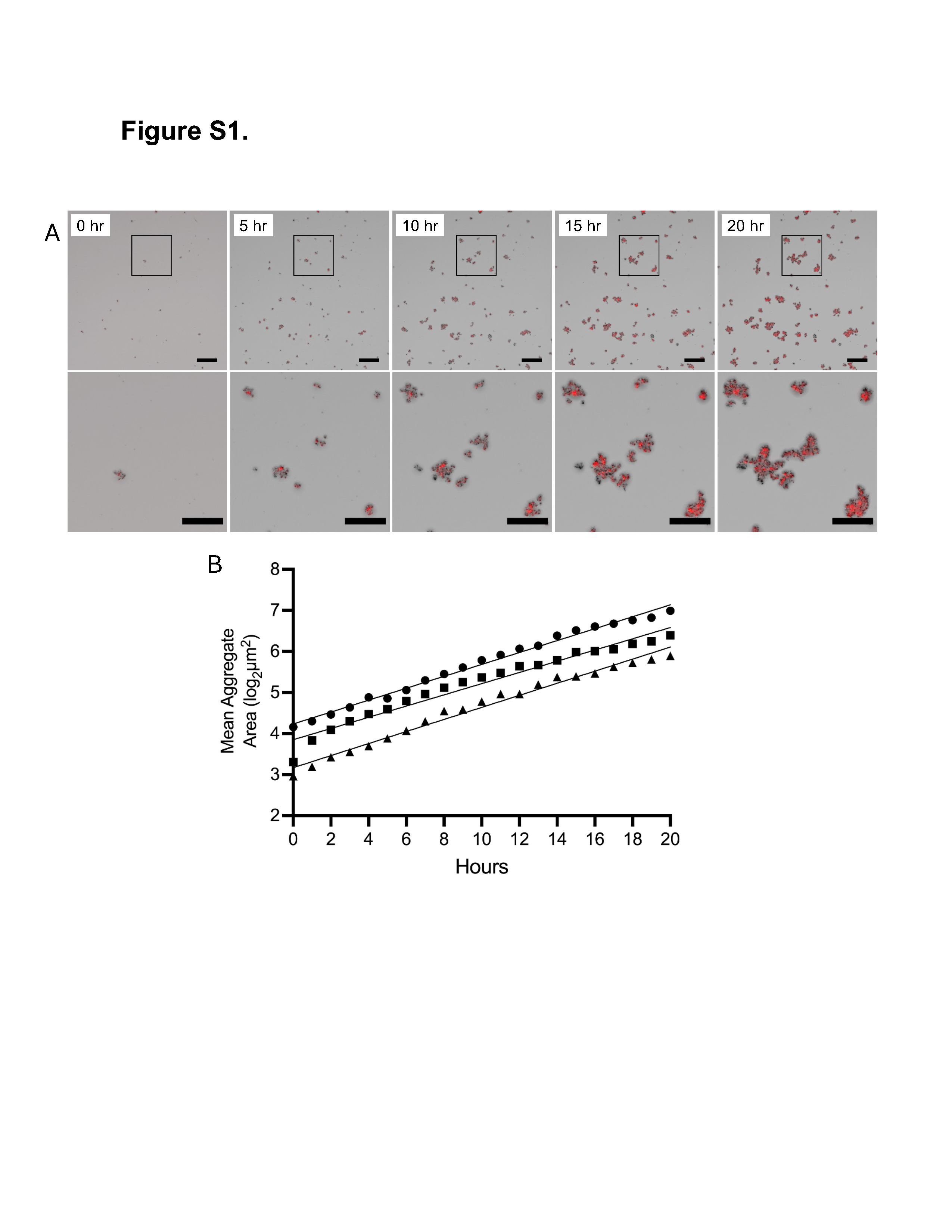

Supplement: Figure S1 — Corynebacterium pseudodiphtheriticum aggregate sizes increases over time. [file jb.00183-25-s0001.tiff]

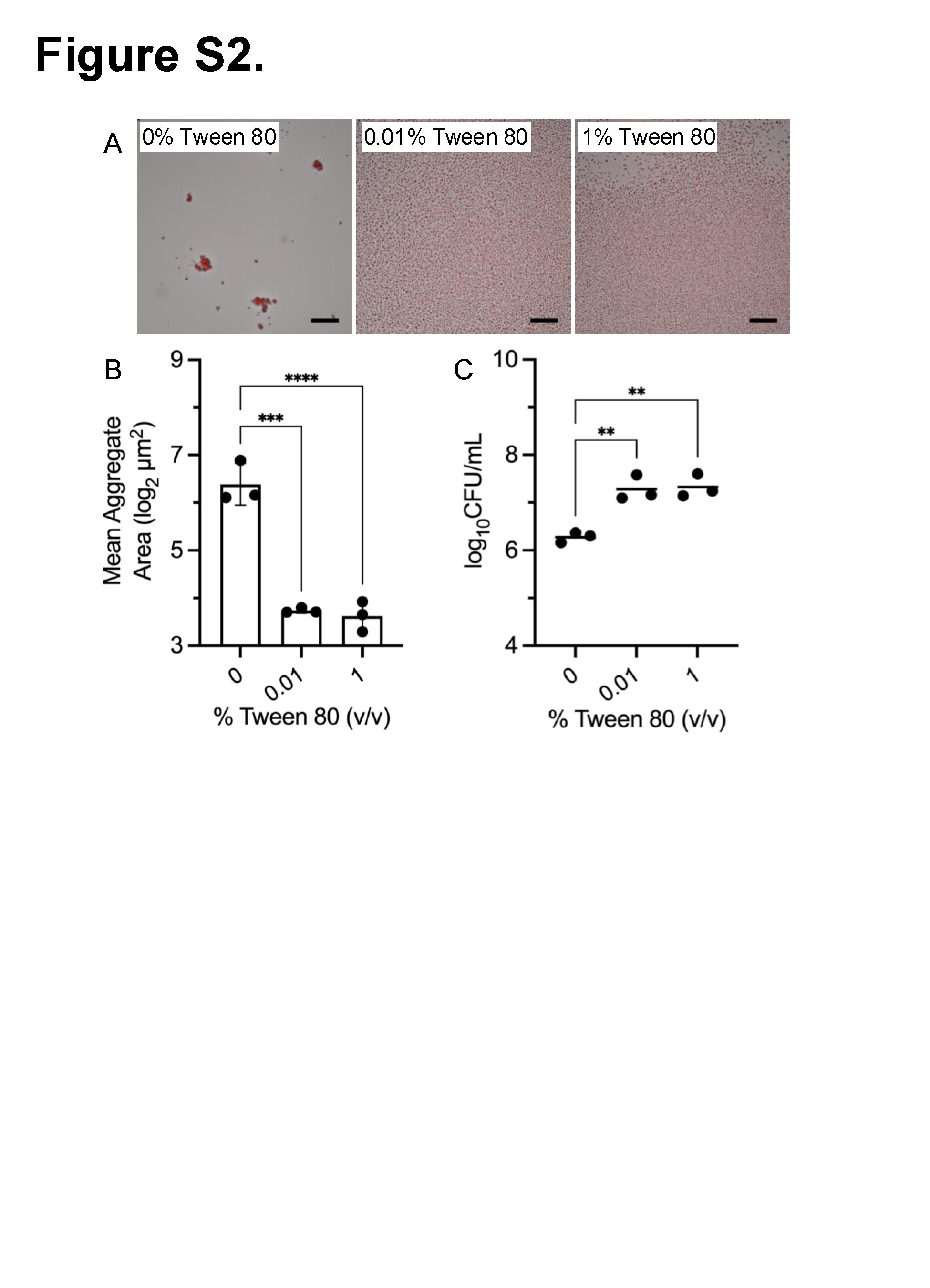

Supplement: Figure S2 — Tween 80 inhibits Corynebacterium pseudodiphtheriticum aggregation. [file jb.00183-25-s0002.tiff]

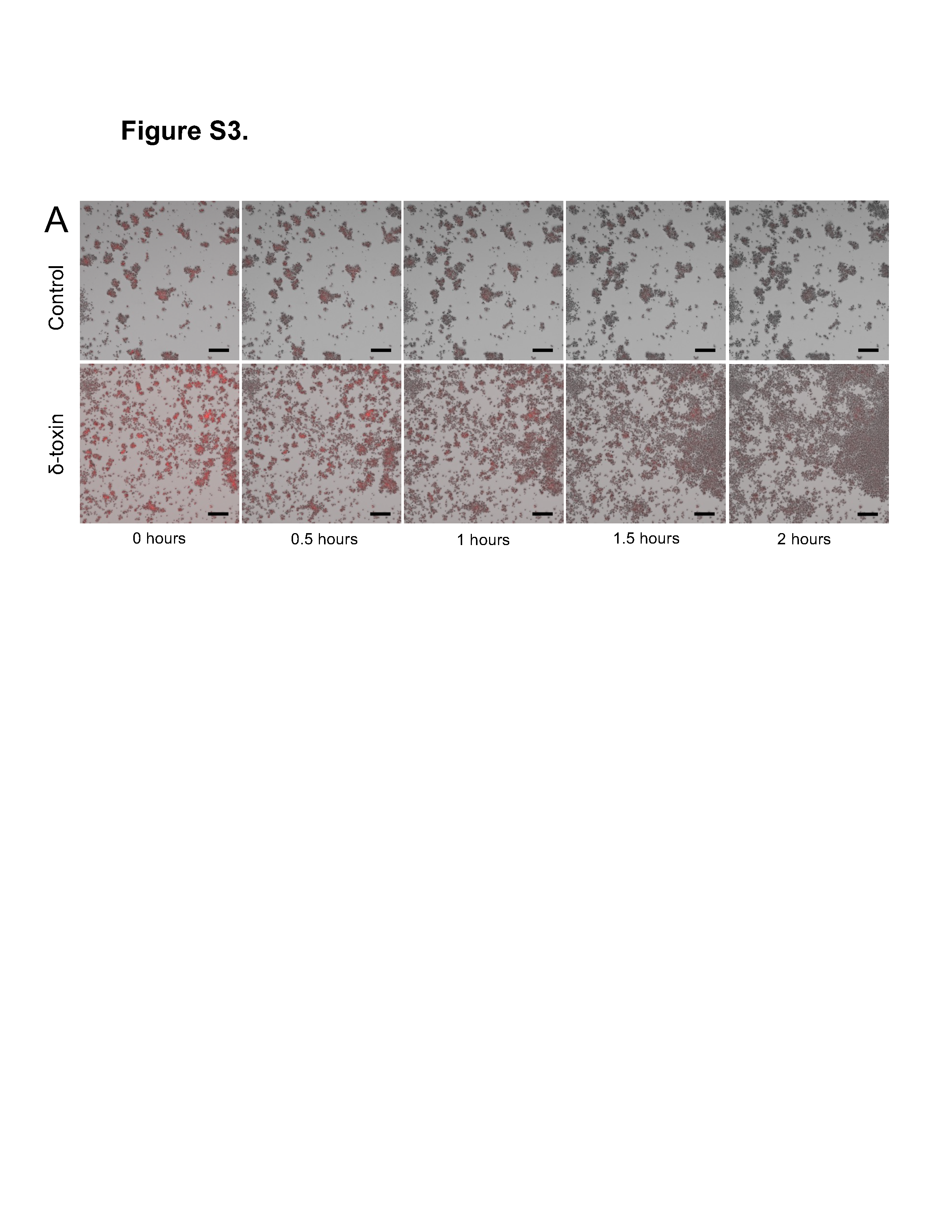

Supplement: Figure S3 — S. aureus phenol-soluble modulins induce Corynebacterium dispersal. [file jb.00183-25-s0003.tiff]

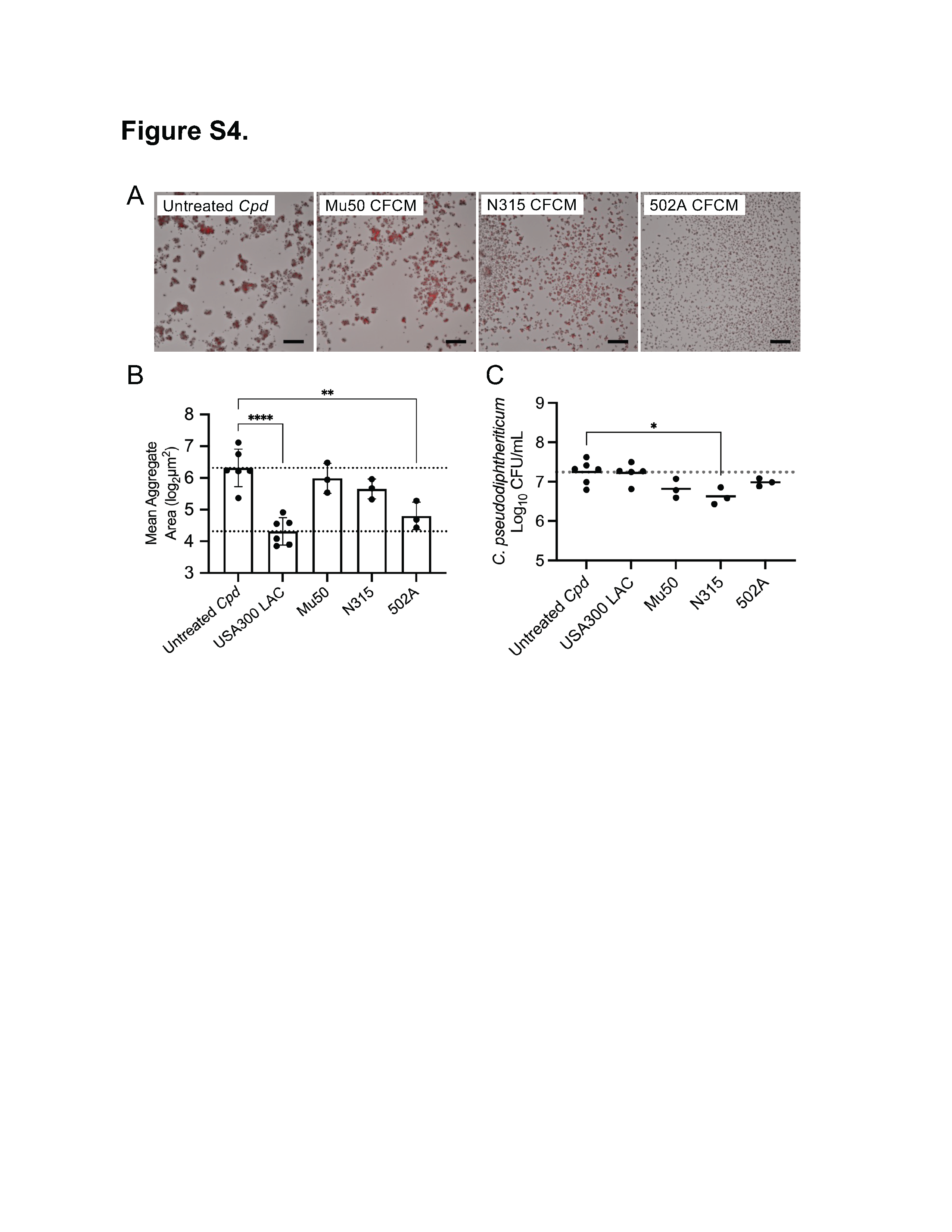

Supplement: Figure S4 — Inhibition of Corynebacterium aggregation is varied amongst Staphylococcus aureus strains. [file jb.00183-25-s0004.tiff]

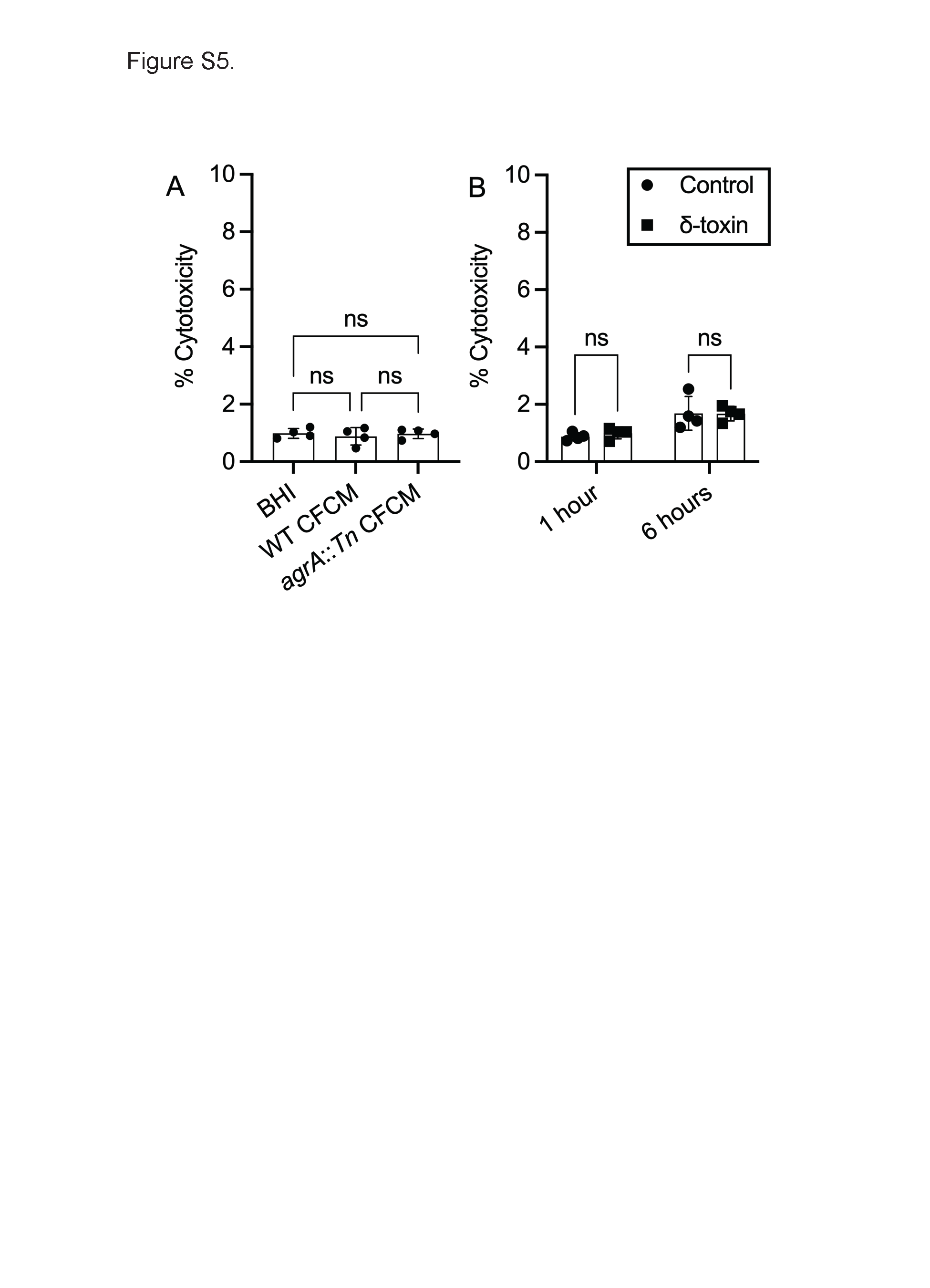

Supplement: Figure S5 — Toxicity of Staphylococcus aureus CFCM and recombinant δ-toxin towards air-liquid interface (ALI) human nasal epithelial cell (HNEC) cultures. [file jb.00183-25-s0005.tiff]
