# Supplementary material for: Staphylococcus aureus phenol-soluble modulins have dispersal and anti-aggregation activity towards corynebacteria
Source: J Bacteriol. 2025 Aug 14;207(9):e00183-25. doi: 10.1128/jb.00183-25 (PMC12445096; doi:10.1128/jb.00183-25)
Supplement: Supplemental legends and Table S1 — Supplemental legends for Fig. S1 to S5, and Movies S1 and S2. Table S1: Bacterial strains. [file jb.00183-25-s0006.pdf]

SUPPORTING INFORMATION

SUPPLEMENTAL FIGURE LEGENDS

Figure S1: *Corynebacterium pseudodiphtheriticum* aggregate sizes increases over time. (A) Timelapse of tdTomato *C. pseudodiphtheriticum* grown over 20-hours in brain-heart infusion broth. 8X magnification of inset on bottom row. Scale bar = 80µm; 40µm for 8X magnification. (B) Quantification of mean aggregate size for A. Lines indicate a linear regression for each biological replicate. *n*=3 biological replicates with 6 technical replicates each. Slopes were not statistically different (*P*=0.2072).

Movie S1: *Corynebacterium pseudodiphtheriticum* grows in aggregate form. (A) tdTomato *C. pseudodiphtheriticum* grown for 20-hours in brain-heart infusion broth.

Figure S2: Tween 80 inhibits *Corynebacterium pseudodiphtheriticum* aggregation. (A) Images of tdTomato-expressing *C. pseudodiphtheriticum* grown for 20 hours in the presence of 0.01% and 1% (v/v) Tween-80 detergent. Scale bar = 80µm. Images are representative of 3 biological replicates with 5 technical replicates each. (B) Quantification of aggregate size for microscopy images in panel A. Statistics: One-way ANOVA with Tukey's multiple comparisons post-hoc test. (C) Colony-forming units for *C. pseudodiphtheriticum* grown for 20 hours in the presence of 0.01% and 1% (v/v) Tween 80 detergent. *n* = 3 biological replicates with 3 technical replicates each. Statistics: One-way ANOVA with Tukey's multiple comparisons post-hoc test.

Figure S3: *S. aureus* phenol-soluble modulins induce *Corynebacterium* dispersal. (A) Timelapse images of *C. pseudodiphtheriticum* exposed to 5 µg/mL recombinant δ-toxin after 20 hours of growth. Images are representative of 3 biological replicates with 2 technical replicates each.

Movie S2: *S. aureus* phenol-soluble modulins directly inhibit and disperse *Corynebacterium pseudodiphtheriticum* aggregation. Timelapse of *C. pseudodiphtheriticum* culture exposed to δ-toxin after 20 hours of growth.

Figure S4: Inhibition of *Corynebacterium* aggregation is varied amongst *Staphylococcus* aureus strains. (A) tdTomato *C. pseudodiphtheriticum* grown for 20-hours in BHI broth with 25% BHI or *S. aureus* CFCM prepared from *S. aureus* cultures grown for 24-hours at 37°C. *n* = 3 biological replicates with 3-6 technical replicates each. (B) Quantification of mean aggregate area from images shown in A. (C) Colony-forming units of *C. pseudodiphtheriticum* from A. *n* = 3-6 biological replicates with 3 technical replicates each. Statistics for B and C: One-way ANOVA with Dunnett's multiple comparisons test. *P<0.05, **P<0.01, ****P<0.0001.

Figure S5: Toxicity of *Staphylococcus aureus* CFCM and recombinant δ-toxin towards air-liquid interface (ALI) human nasal epithelial cell (HNEC) cultures. Cytotoxicity measured through LDH release from ALI HNEC cultures into the basolateral media for (A) 10% brain-heart infusion broth or *S. aureus* CFCM from wild-type or *agrA*::*Tn* strains and (B) 1- and 6-hour exposure to 5 µg/mL recombinant δ-toxin.

Statistics for A: One-way ANOVA with Tukey's multiple comparisons post-hoc test. *n* = 4 biological replicates. Statistics for B: Two-way ANOVA with Sidak's multiple comparisons post-hoc test. *n* = 4 biological replicates.

Table S1. Bacterial Strains

**Strain name**	**Description**	**Reference**
Staphylococcus aureus **strains**		
S. aureus LAC 13c	USA300 CA-MRSA, erm^S	(32)
S. aureus 502A	Human nasal colonizing isolate	(65)
S. aureus N315	Hospital-acquired MRSA	(66)
S. aureus Mu50	Hospital-acquired MRSA	(66)
S. aureus LAC 13c *psmα-*	USA300 LAC Δpsm*a*1-4	(41)
S. aureus LAC 13c *psmα-/hld*^{ATT}	USA300 LAC Δ*psm*α1-4 δATG-ATT	(41)
Corynebacterium **strains**		
Corynebacterium pseudodiphtheriticum + pJOE7706.1-tdtomato	Sinus clinical isolate expressing pJOE7706.1 with tdtomato inserted at BsrGI and BamHI sites	(31)

Corynebacterium propinquum	Sinus clinical isolate	
Corynebacterium pseudodiphtheriticum ATCC	ATCC strain 153, Lehmann and Neumann	(67)
Corynebacterium amycolatum	Strain SK46, skin isolate	(68)
Corynebacterium accolens	ATCC strain 49276, Neubauer	(69)
**Nebraska Transposon Mutant Library *S. aureus* strains**		
S. aureus agrA::Tn	Transposon Mutant NE1532 (SAUSA300_1992)	(32)
S. aureus sigB::Tn	Transposon Mutant NE1109 (SAUSA300_2022)	(32)
S. aureus sarA::Tn	Transposon Mutant NE1193 (SAUSA300_0605)	(32)
S. aureus saeR::Tn	Transposon Mutant NE1622 (SAUSA300_0691)	(32)

S. aureus aur::Tn	Transposon Mutant NE163 (SAUSA300_2572)	(32)
S. aureus sspA::Tn	Transposon Mutant NE1506 (SAUSA300_0951)	(32)
S. aureus sspB::Tn	Transposon Mutant NE934 (SAUSA300_0950)	(32)
S. aureus splC::Tn	Transposon Mutant NE1098 (SAUSA300_1756)	(32)
S. aureus splF::Tn	Transposon Mutant NE1764 (SAUSA300_1753)	(32)
S. aureus geh::Tn	Transposon Mutant NE1775 (SAUSA300_0320)	(32)
S. aureus lip::Tn	Transposon Mutant NE338 (SAUSA300_2603)	(32)
S. aureus pmtB::Tn	Transposon Mutant NE1188 (SAUSA300_1912)	(32)
S. aureus pmtC::Tn	Transposon Mutant NE1908 (SAUSA300_1911)	(32)

References:

65. Parker D, Narechania A, Sebra R, Deikus G, LaRussa S, Ryan C, Smith H, Prince A, Mathema B, Ratner AJ, Kreiswirth B, Planet PJ. 2014. Genome sequence of bacterial interference strain *Staphylococcus aureus* 502A. Genome Announc 2. https://doi.org/10.1128/genomeA.00284-14

66. Kuroda M, Ohta T, Uchiyama I, Baba T, Yuzawa H, Kobayashi I, Cui L, Oguchi A, Aoki K, Nagai Y, et al. 2001. Whole genome sequencing of meticillin-resistant *Staphylococcus aureus*. The Lancet 357:1225–1240. https://doi.org/10.1016/S0140-6736(00)04403-2

67. Altenburger P, KaMPFER P, Akimov VN, Lubit W, Busse H-J. 1997. Polyamine distribution in actinomycetes with group B peptidoglycan and species of the genera *Brevibacterium, Corynebacterium,* and *Tsukamurella*. Int J Syst Bacteriol 47:270–277. https://doi.org/10.1099/00207713-47-2-270

68. Peterson J, Garges S, Giovanni M, McInnes P, Wang L, Schloss JA, Bonazzi V, McEwen JE, Wetterstrand KA, Deal C, et al. 2009. The NIH human microbiome project. Genome Res 19:2317. https://doi.org/10.1101/gr.096651.109

69. Neubauer M, Šourek J, Rýc M, Boháček J, Mára M, Mňuková J. 1991. *Corynebacterium accolens* sp. nov., a gram-positive rod exhibiting satellitism, from clinical material. Syst Appl Microbiol 14:46–51. https://doi.org/10.1016/S0723-2020(11)80360-7
